# Impact of microbiota on breast cancer hormone therapy

**DOI:** 10.15698/cst2023.03.277

**Published:** 2023-03-13

**Authors:** Safae Terrisse, Laurence Zitvogel, Guido Kroemer

**Affiliations:** 1 Medical Oncology, Hôpital Saint-Louis, Paris, France.; 2 INSERM U1015, Equipe Labellisée - Ligue Nationale contre le Cancer, Villejuif, France.; 3 University Paris Saclay, Gif-sur-Yvette, France.; 4 Gustave Roussy, ClinicObiome, Villejuif, France.; 5 Center of Clinical Investigations in Biotherapies of Cancer (CICBT) 1428, Villejuif, France.; 6 Equipe labellisée par la Ligue contre le Cancer, Université de Paris Cité, Sorbonne Université, Institut Universitaire de France, Inserm U1138, Centre de Recherche des Cordeliers, Paris, France.; 7 Metabolomics and Cell Biology Platforms, Gustave Roussy Comprehensive Cancer Institute, Villejuif, France.; 8 Institut du Cancer Paris CARPEM, Department of Biology, Hôpital Européen Georges Pompidou, AP-HP, Paris, France.

**Keywords:** breast cancer, hormone therapy, microbiota, immunity

## Abstract

Recent observations indicate that the pathogenesis and prognosis of hormone-receptor breast cancer is not only dictated by the properties of the malignant cells but also by immune and microbial parameters. Thus, the immunosurveillance system retards the development of hormone-positive breast cancer and contributes to the therapeutic efficacy of estrogen receptor antagonists and aromatase inhibitors. Moreover, the anticancer immune response is profoundly modulated by the local and intestinal microbiota, which influences cancer cell-intrinsic signaling pathways, affects the composition and function of the immune infiltrate present in the tumor microenvironment and modulates the metabolism of estrogens. Indeed, specific bacteria in the gut produce enzymes that affect the enterohepatic cycle of estrogen metabolites, convert estrogens into androgens or generate estrogen-like molecules. The knowledge of these circuitries is in its infancy, calling for further in-depth analyses.

## INTRODUCTION

Immunotherapy is now part of standard clinical practice in cancer therapy. Although historically disappointing, immunotherapy in breast cancer (BC) has recently gained momentum. Thus, treatment with pembrolizumab, an immune checkpoint inhibitor (ICI) targeting PD-1 (programmed death-1), appears to be effective against early stage and advanced triple negative breast cancer (TNBC) according to the randomized Phase III trials KEYNOTE-522 [1] and KEYNOTE-355 [2], respectively. These trials led to the first FDA approval of immunotherapy for the treatment of TNBC.

Nevertheless, hormone receptor-positive (HR^+^) breast cancer (BC) is still lagging behind in the development of immunotherapy. Historically, HR^+^ BC has been mostly treated by hormone therapies (i.e., estrogen receptor blockade or aromatase inhibitors that suppress estrogen biosynthesis) and conventional chemotherapies (e.g., anthracycline and taxanes) and considered to be primarily immunoresistant [3–5]. This idea has, however, been attenuated by the observation that HR^+^ BC can be under immunosurveillance. For example, ductal carcinomas in situ (DCIS), which mostly are HR^+^ [6], have a particularly low incidence of recurrence after surgical removal when the ratio of cytotoxic T lymphocytes over regulatory T cells (CTL/Treg ratio) infiltrating the normal breast tissue indicates a favorable immune tonus [7]. Moreover, the effects of hormone therapy against HR^+^ BC involves a strong immune component [8–10].

The composition of local and intestinal microbiota, as well as its therapy-induced modifications affect the response to anti-cancer treatments (i.e., chemotherapy, targeted therapy and immunotherapy) [11, 12]. In addition, the gut microbiota strongly influences the therapeutic response of hormone dependent cancers [13, 14]. Since androgen deprivation therapy (ADT) of prostate cancer must induce an anticancer immune response to be efficient, and since the immune tonus is influenced by intestinal commensals, dysbiosis may have a negative impact on the efficacy of ADT [13]. Moreover, intestinal bacteria that produce androgens as well as bacteria that degrade drugs used for ADT, can interfere with the efficacy of ADT [14, 15]. By analogy to prostate cancer ADT, we surmise that BC hormone therapy might be influenced by the microbiota, and this is the topic of the present mini-review.

Here, we summarize accumulating evidence indicating that the microbiota modulates the efficacy of hormone therapy against HR^+^ BC. This modulation involves two distinct facets. On one hand, the microbiota present in the gut or in malignant tissues affects the immune tonus, thus attenuating or enhancing the anticancer immune response stimulated by hormone therapy. On the other hand, bacteria reportedly can synthesize, recycle or destroy estrogens in the gut, thereby affecting the concentrations of cancer cell-supportive hormone. We will discuss the clinical implication of these findings and detail promising strategies for intervening on the microbiota.

## IMMUNOSURVEILLANCE OF HR^+^ BC

### Inflammation in the pathogenesis of HR^+^ BC

HR^+^ BCs are commonly described as immunologically cold tumors, with low abundance of tumor-infiltrating lymphocytes (TILs) [16] and scarce expression of PD-L1 (programmed cell death protein-ligand 1) [17, 18]. Nonetheless, mounting evidence suggests that immunity and inflammation may be relevant to HR^+^ BC biology [19]. Thus, it turned out that, in a mouse model, medroxyprogesterone acetate (MPA, a progesterone analogue) and 7,12-dimethylbenz[a]anthracene (DMBA, a DNA damaging agent)-induced HR^+^ BCs are under strong immunosurveillance [20]. MPA/DMBA-induced mammary carcinomas resemble human luminal B HR^+^ HER2^-^ (human epidermial growth factor 2 negative) BC, in particular with respect to their transcriptome; as well as with respect to limited immune infiltration and low responsiveness to PD-1 blockade [20]. Nonetheless, MPA/DMBA-induced oncogenesis and tumor progression is accelerated in the context of natural killer (NK) and T cell defects, demonstrating that MPA/DMBA-induced tumors are under immunosurveillance [20].

Leukocytes do not only mediate immune responses necessary for immunosurveillance but are also involved in procarcinogenic inflammation, likely contributing to the protumoral effects of obesity, which is the most prevalent pathological condition affecting humanity. Indeed, obesity promotes a state of chronic inflammation leading to the local accumulation of macrophages, the production of cytokines (such as CCL2 and IL-1β), as well as immunosuppression of T lymphocytes [21]. The obesity-associated accumulation of necrotic adipocytes surrounded by macrophages forming crown-like structures (CLS) in breast tissue has been associated with poor prognosis [22, 23]. Of note, high-fat diet (HFD), which causes obesity in mice, accelerated MPA/DMBA-induced carcinogenesis and shortened overall survival, while alternative day fasting decelerated the process and extended overall survival [20]. This observation appears concordant with the fact that obesity is a major risk factor for BC development, progression and therapeutic response [24–25]. This is most clearly shown for postmenopausal HR^+^ BC, though less established for TNBC and human epidermal growth factor-2-positive (HER2^+^) BC [26]. Indeed, overabundant white adipose tissue expresses enzymes that catalyze estrogen biosynthesis [27–28]. Thus, compared to that from lean BC carriers, the breast tissue from obese women with BC contains elevated levels of aromatase, a key enzyme in estrogen biosynthesis [29], possibly compromising the therapeutic effects of hormone therapy [30].

### Immunomodulation by endocrine therapy

Oophorectomy can postpone the death of mice after intraperitoneal injection of ovarian cancer cells (ID8-*Defb29/Vegfa* cells, which are not responsive to estrogens *in vitro*), and this effect is lost in *Rag1*^*-/-*^ mice (which lack B and T cells), pointing to the possible implication of the immune system in endocrine therapy [31]. In the MPA/DMBA-induced mouse mode of HR^+^ BC, genetically-induced estrogen receptor deficiency leads to a delay in cancer development, and this effect could be phenocopied by continuous treatment with the estrogen receptor antagonist tamoxifen. However, the tamoxifen-mediated delay in cancer development and progression was only observed in immunocompetent, not in immunodeficient (*Rag2*^−/−^*Il2rg*^−/−^) animals, underscoring the importance of immunosurveillance for the anticancer efficacy of estrogen pathway blockade [20].

Most immune cells express estrogen receptors (ER), making these cells sensitive to estrogens and their modulators [32, 33]. Thus, beyond their direct cell-autonomous effects on HR^+^ BC cells, tamoxifen and aromatase inhibitors may mediate effects on the immune system. Indeed, tamoxifen reduces the infiltration by, and immunosuppressive activity of, myeloid-derived suppressor cells (MDSCs) in BC [31]. Tamoxifen has been suggested to block M2 polarization of the microglia in the brain, thereby inhibiting BC brain metastasis [34]. The aromatase inhibitor fulvestrant significantly reduced macrophage and neutrophil neutralization of human BC transplanted into T cell-deficient mice [35]. Another aromatase inhibitor, anastrozole, inhibits the differentiation of naïve T cells into Treg, promoted immunostimulatory cytokines such as IFN-γ and IL-12, and decreased immunosuppressive cytokines such as IL-4 and IL-10 [36].

In accord with this preclinical literature, in two independent cohorts of patients receiving neoadjuvant aromatase inhibitors, the CTL/Treg ratio was significantly increased and Forhead box P3 (FOXP3)^+^ Tregs decreased in responders but not non-responders after estrogen deprivation [8, 9]. Moreover, the abundancy of TILs constitutes a predictive biomarker for tamoxifen responses in premenopausal breast cancer [10]. Thus, estrogen-targeted therapies have an immunomodulatory capacity, which might be enhanced by immunotherapy. Different clinical strategies are currently under evaluation, such as the combination of an HDAC inhibitor (vorinostat) and a PD-1 inhibitor (pembrolizumab) with tamoxifen (NCT02395627). Moreover, trials are addressing the efficacy of therapeutic vaccination against HR^+^ BC (NCT02229084, NCT00925548).

In summary, it appears that the immune system can control HR^+^ BC to some extent and that hormone therapy mediates at least part of its effects on HR^+^ BC by dampening protumorigenic inflammation, as well as by restoring immunosurveillance.

## CONTRIBUTION OF MICROBIOTA TO CARCINOGENESIS AND PROGNOSIS OF HR^+^ BC

### Intestinal microbiota

Some data suggest a link between the gut microbiota and breast cancer risk and prognosis [37]. In one study, stool samples from 31 patients with early BC were examined by 16S rRNA sequencing and RT-qPCR for genes specific for different bacterial families or species [38]. The authors concluded that patients with stage II-III (versus stage I) BC were enriched for *Clostridium leptum* and *Clostridium coccoides*, which both belong to the *Firmicutes* phylum and express β-glucuronidases that may favor the reabsorption of free estrogen [38]. However, circulating estrogen levels were not measured in this study to support this conjecture.

More recently, shotgun metagenomics was used to determine the composition of the fecal microbiota in 121 specimens from 76 early, mostly RH^+^ BC patients [39]. The study corroborated the previously suggested deleterious role in BC outcome of Clostridiaceae family members (*Clostridium citroniae, Clostridium bolteae, Clostridium clostridioforme, Clostridium symbosium, Clostridium aldenese, Clostridium hathewayi, Clostridium asparagiforme*) as also seen in other malignancies (kidney, lung) [40, 41]. In contrast, *Eubacterium rectale, Methanobrevibacter smithii, Coprococcus comes, Coprococcus catus* and Actinobacteria (*Collinsella aerofaciens*) were associated with healthy status, as well as good prognosis BC (stage I or absence of lymph node involvement) [39] in line with previous reports in patients with kidney cancer or melanoma treated with immune checkpoint inhibitors [41, 42]. *Akkermansia muciniphila*, which is known to protect against metabolic syndrome and obesity [43] and stimulates anticancer immune responses associated with favorable prognosis in lung and kidney cancer patients [12, 40, 44] was found to be associated with small BC tumor size (pT1). Of note, 55% of women with BC lacked detectable *A. muciniphila*, as also observed in the healthy population, but consistent with the association of BC with type 2 diabetes and obesity [39]. In immunocompetent mice orally gavaged with BC female stools, fecal microbiota transplants (FMT) containing *Eubacterium* species (*Eubacterium rectale, Eubakterium eligens, Eubakterium ventriosum*) or *C. aerofaciens* reduced the growth of syngeneic AT3 BC cells, suggesting that the intestinal microbiota can indeed modulate BC immunosurveillance [39].

**Figure 1:**
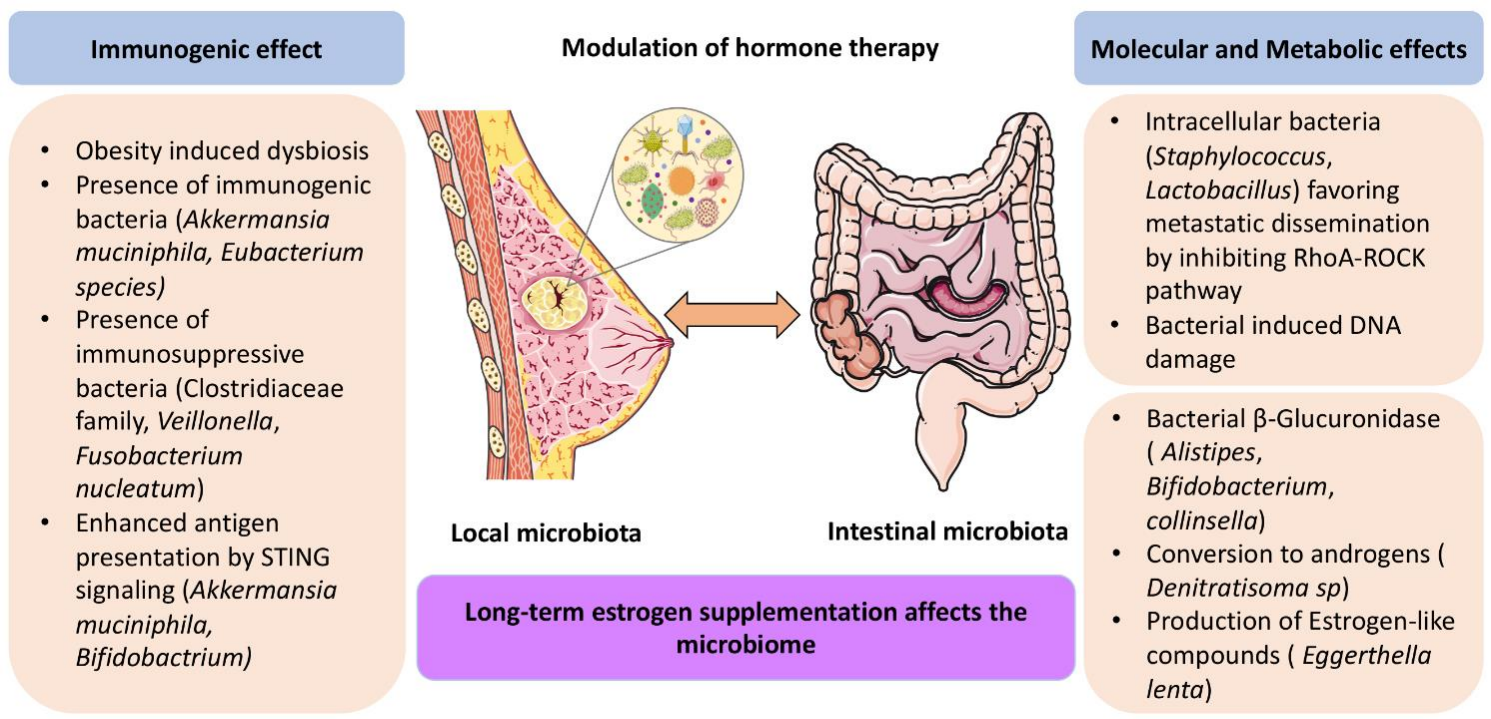
FIGURE 1: Potential mechanisms explaning the effects of the local and intestinal microbiota on hormone receptor-positive breast cancer carcinogenesis and sensitivity to hormone therapies. For details consult text.

In sum, it appears that the intestinal microbiota is altered in advanced BC and that alterations in the microbiota may affect BC progression (**Figure 1**). Of note, chemotherapy can affect the BC-associated microbiota, shifting it to a more favorable composition [39]. Whether this is a consequence of tumor size reduction or vice versa or explains (some of) the antineoplastic effect(s) of chemotherapy remains to be determined.

### Local microbiota

Bacteria and fungi are locally present in several types of cancers (i.e., breast, lung, melanoma, pancreas) as described in large-scale studies by Ravid Straussman's group [45, 46]. Earlier research identified a discriminant signature in the three breast cancer subtypes (HR^+^ BC, HER2^+^ BC, TNBC), HR^+^ BC showing the most diverse local microbiome, whereas TN was characterized by a high prevalence of *Fusobacterium nucleatum* [47]. Intratumoral microbes may favor oncogenesis [48, 49] by several putative mechanisms: local genotoxicity by direct DNA damage [50], activation of oncogenic pathways (e.g., TLR/β catenin pathway activation by *F. nucleatum* in colorectal cancers) [51], promotion of immune escape or chronic inflammation [52] or induction of chemoresistance mechanism (e.g., via induction of autophagy in colorectal cancer [53]. However, the literature on the specific contribution of the local microbiota to BC pathogenesis is scarce. Specific intracellular bacteria (*Lactobacillus, Staphylococcus* and *Streptococcus*) have been shown to inhibit the RhoA-ROCK pathway, thereby increasing the resistance of BC cells to mechanical stress and favoring their metastatic dissemination [54]. In this paradigm, eradication of these bacteria by suitable antibiotics had no effect on the growth of the primary tumor, but did reduce the capacity of BC cells to metastasize [54, 55].

Beyond its direct effects on the oncogenic potential of malignant cells, the intratumoral microbiota may modulate local immunity through dual effects that either foster an immunosuppressive tumor environment or support anticancer immunity [56]. Microbial peptides from intracellular bacteria may be presented by the MHC class I or II molecules on the surface of tumor cells, thus offering a target for CTL and CD4+ T cells respectively [57]. Moreover, bacteria can trigger pattern recognition receptors. For example, *Bifidobacterium*, a bacterial family naturally present in the human gut, has been found in malignant tissues to activate the innate STING signaling pathway, thereby improving antigen presentation by dendritic cells [58]. *A. muciniphila* also activates the STING pathway to enhance the secretion of Type 1 IFN and hence reshape the tumor microenvironment [56]. Whether these findings also apply to BC remains to be determined (**Figure 1**).

The ultimate conundrum is the source of these intratumoral microbes. A study on canine mammary tumors reported the existence of the same species of *Bacteroides* in the tumor microbiota as in the mouth and the gut, suggesting bacterial migration along the intestinal tract and to distal malignant tissue via the blood stream [59]. However, at this point, other routes (such as ascending bacterial contamination of milk ducts) cannot be excluded.

## IMPACT OF MICROBIOTA ON THE EFFICACY OF BC HORMONE THERAPY

Bacteria present in the gut have a major impact on the enterohepatic circulation of estrogens. Estradiol is conjugated in the liver by glucuronyltransferases into estradiol glucuronide and excreted via bile into the gut, where it can be deconjugated by bacterial β-glucuronidases and then be reabsorbed [60]. Bacteria that produce β-glucuronidase include *Alistipes, Bacteroides, Bifidobacterium, Collinsella, Edwardsiella, Faecalibacterium genera*, and *Lactobacillus* and *Roseburia species* [61, 62]. However, there are no systematic studies on the implication of such bacteria in the pathogenesis of HR2^+^ BC. Of note, specific bacteria (such as the betaproteobacterium *Denitratisoma* sp. strain DHT3) can convert estrogens into androgen [63]. Whether such bacteria endowed with the conversion of female into male sex hormone are contained in the human gut remains to be determined. Reportedly, human feces (especially from female subjects) contain bacteria such as *Peptostreptococcus productus* SECO-Mt75m3 and *Eggerthella lenta* SECO-Mt75m2, which produce estrogen-like compounds such as enterodiol (ED) and enterolactone (EL) [64]. Hence, it can be speculated, yet remains to be demonstrated, that the abundance of such bacteria affects the development of HR^+^ BC as well as the response of HR^+^ BC to hormone therapy.

Long-term estrogen supplementation of mice affects the composition of the gut microbiota (with a decrease of *A. muciniphila*), as well as estrogen metabolism (due to a reduction in β-glucuronidase activity) in the murine gut, suggesting that estrogen inhibition should affect the intestinal microbiota as well [65]. However, at this point, it has not been reported that estrogen receptor antagonists and aromatase inhibitors would increase the intestinal abundancy of *A. muciniphila*, which might be expected to have favorable effects on anticancer immune responses [40, 44, 66, 67]. In patients with endocrine-resistant HR^+^ BC escaping from adjuvant aromatase inhibitor therapy, shifts in the fecal microbiota were observed compared to patients who continued to respond. In particular, bacteria belonging to the *Veillonella* genus were overabundant in women with endocrine-resistant HR^+^ BC [68]. Of note, *Veillonella* species have been associated with poor prognosis if present in the gut of patients treated with CAR-T cells [69] or in the tumor microbiota of lung cancer patients [70]. However, its causal implication in HR^+^ BC responses to hormone therapy remains elusive.

## CONCLUSIONS

As summarized in this review, HR^+^ BC is similar to other cancers with respect to its broad relationship to pro-inflammatory circuitries (which are oncogenic, explaining the epidemiological association of HR^+^ BC with obesity), immunosurveillance (which limits HR^+^ BC oncogenesis at least in experimental models) and the local and remote (mostly intestinal) microbiota. The microbiota may impact the pathogenesis of HR^+^ BC at multiple levels, (i) locally by affecting malignant cell-intrinsic properties, (ii) locally by modulating the tumor microenvironment, and (iii) systemically by long-distance effects emanating from the gut microbiota that can be mediated by metabolic, inflammatory and immune circuitries [71]. At this latter level, it appears that the intestinal microflora potentially mediates the synthesis of estrogen receptor agonists, destroys estrogens and modulates the enterohepatic circulation of estrogens, hence influencing the overall estrogen tonus.

In this context, it appears that the preponderant treatment modality applied to HR^+^ BC, which consists in the administration of estrogen receptor antagonist or that of aromatase inhibitors, has profound effects on immune cells (which express estrogen receptors), meaning that (part of) their therapeutic efficacy may transcend the cancer cell-autonomous action of such hormone therapies. Indeed, in preclinical models, hormone therapies appear to be more efficient in the presence of an intact immune system, a hypothesis that is compatible with some epidemiological observations such as the correlation between therapeutic efficacy and a favorable CTL/Treg ratio among TILs present in BC. Since the intestinal microbiota plays a major role in shaping the inflammatory and immune tonus within tumors [72], it can be expected that the composition of the microflora as well as its functional state (i.e., eubiosis versus dysbiosis) should impact the outcome of hormone therapy. In addition, it appears plausible, yet remains to be demonstrated, that a microbiota-driven increase in estrogen levels or the enzymatic destruction of orally administered hormone therapies may impact the pharmacology of hormone therapy.

Beyond theoretical considerations, it will be important to understand how the local and intestinal microbiota can be modified for improving the clinical outcome of HR^+^ BC treatments. Future investigation may lead to the identification of specific favorable bacteria that improve the hormonal, metabolic and immune control of HR^+^ BC. In this, case, prebiotics (compounds that expand useful microbes), probiotics (specific microbial species) and postbiotics (the products including the metabolites of such microbes) might be useful [73]. Similarly, future research might identify harmful microbes that should be selectively eliminated by antibiotics, lysed by phages or held in check by the host immune system, for instance as a result of vaccination campaigns [72]. However, it is also possible that, instead of individual microbes, systemic properties of the microbial ecosystem must be manipulated to improve the homeostatic control of the diseased tissue [11, 74]. Future research should actively explore these possibilities.

## Abbreviations:

ADT: – androgen deprivation therapy;
BC: – breast cancer;
CTL: – cytotoxic T lymphocyte;
DMBA: – 7,12-dimethylbenz[a]anthracene;
HR^+^: – hormone-receptor positive;
MPA: – medroxyprogesterone acetate;
TIL: – tumor-infiltrating lymphocyte;
TNBC: – triple negative BR;
Treg: – regulatory T cell.
